# 21-Year-Old Pregnant Woman with MODY-5 Diabetes

**DOI:** 10.1155/2017/6431531

**Published:** 2017-10-15

**Authors:** Anastasia Mikuscheva, Elliot McKenzie, Adel Mekhail

**Affiliations:** Department of Gynecology and Obstetrics, Dunedin University Hospital, Dunedin, New Zealand

## Abstract

The term “Maturity-Onset Diabetes of the Young” (MODY) was first described in 1976 and is currently referred to as monogenic diabetes. There are 14 known entities accounting for 1-2% of diabetes and they are frequently misdiagnosed as either type 1 or type 2 diabetes. MODY-5 is an entity of monogenic diabetes that is associated with genitourinary malformations and should be considered by obstetricians in pregnant women with a screen positive for diabetes, genitourinary malformations, and fetal renal anomalies. Correct diagnosis of monogenic diabetes has implications on managing patients and their families. We are reporting a case of a 21-year-old pregnant woman with a bicornuate uterus, fetal renal anomalies, and a family history of diabetes that were suggestive of a MODY-5 diabetes.

## 1. Introduction

Monogenic beta-cell diabetes is thought to be responsible for approximately 2% of all diabetes cases diagnosed before the age of 45 years [1]. Approximately 80% of cases are misdiagnosed as either type 1 or type 2 diabetes, reflecting lack of physician awareness and/or access to genetic testing [2]. Clues to the diagnosis of monogenic forms of diabetes include lack of typical characteristics of type 1 diabetes (no pancreatic autoantibodies, low or no insulin requirement five years after diagnosis, persistence of stimulated C-peptide of 4200 pmol/L, absence of diabetic ketoacidosis) or type 2 diabetes (lack of obesity, hypertension, dyslipidemia), in the presence of a strong family history [1].

Renal cysts and diabetes syndrome (RCAD) or Maturity-Onset Diabetes of the Young type 5 (MODY-5) is a form of monogenic diabetes caused by a mutation in the gene coding for the transcription factor hepatocyte nuclear factor 1-beta (HNF1B) [3]. HNF1B is critical for the development of the kidney and pancreas. In humans, mutations in HNF1B lead to congenital anomalies of the kidney and urinary tract, pancreas atrophy, pancreatic endocrine and exocrine deficiency, and genital malformations. [4]. The majority of HNF1 mutation carriers have extrarenal phenotypes with diabetes being the most common.

The gene was first described as causing Maturity-Onset Diabetes of the Young type 5 [5]; however, it is more commonly associated with renal disease. Diabetes usually presents in early adulthood with a median age of 20 years (range 15 days to 61 years) and frequently requires insulin treatment [6]. Renal abnormalities are frequently detected on antenatal ultrasound scans from as early as 17 weeks of gestation. Patients with a HNF1B mutation have renal function that ranges from normal to dialysis dependent or transplanted [7].

Genital tract malformations in HNF1B disease were first reported by Lindner et al. [8] in 1999; subsequently, a range of uterine malformations have been described that include bicornuate uterus, uterus didelphys, rudimentary uterus, and vaginal atresia [9].

It is important to establish in a subject with a uterine and renal abnormality whether they have a HNF1B mutation because of the 50% chance of having transmitted this to any of their existing children or to any future pregnancies. Their children will be identified as being at increased risk of the development of any of the features of the HNF1B phenotype [10].

## 2. Case Report

**Figure 1:**
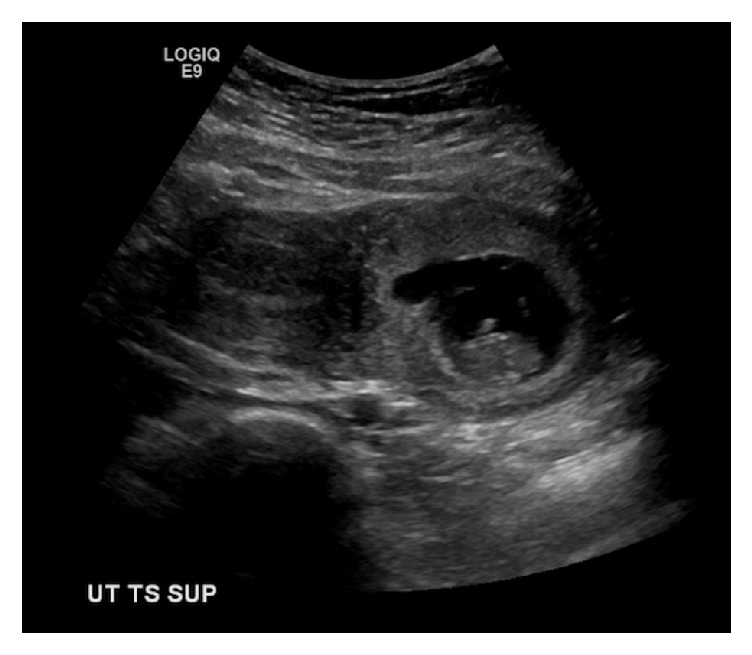
Bicornuate uterus at 9 + 5-week dating scan.

**Figure 2:**
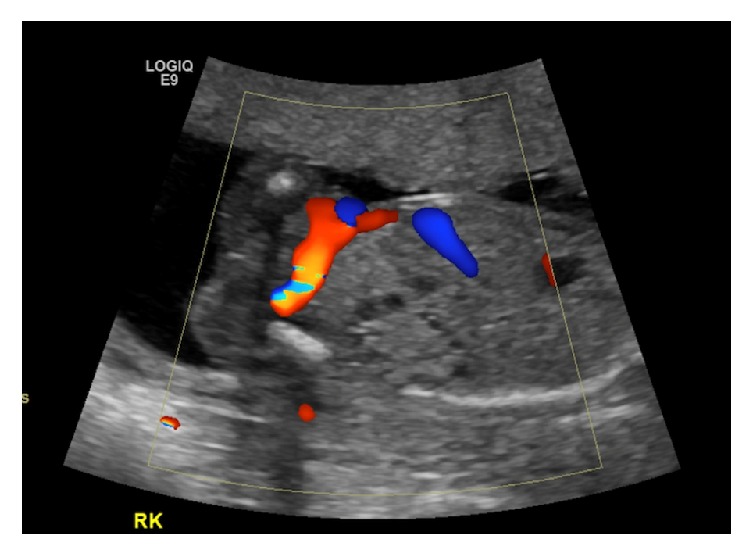
Multicystic dysplastic right kidney in the fetus at 19-week GA.

**Figure 3:**
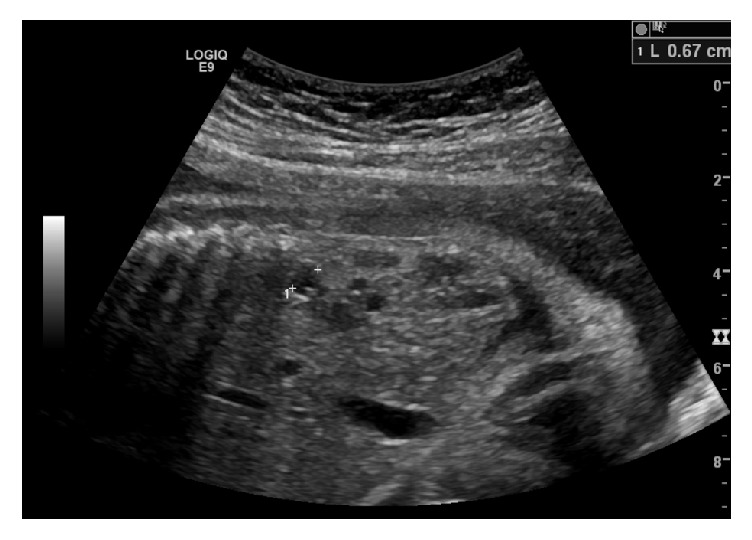
Multicystic dysplastic right kidney at 27 + 6 weeks.

Miss A. was a 21-year-old fit and well woman without a prior documented significant medical or surgical history, who was referred to our antenatal clinic by her midwife because a bicornuate uterus was discovered on the dating scan (Figure 1) of her spontaneously conceived first pregnancy. Physical examination was normal. Full blood count and liver function tests were normal. She was negative for HIV, hepatitis B, Chlamydia, and syphilis. An HbA1C, routinely done in New Zealand for diabetes screening, was mildly elevated at 41 mmol/mol. Because of this result the patient was referred to the combined endocrine clinic and blood sugar monitoring was commenced. She had persistent hyperglycemia and investigations to exclude type I diabetes were performed. The anti-GAD antibodies and Islet Cell Cytoplasmic autoantibodies came back negative. She was started on insulin treatment as blood sugars were higher than 8 mmol/l on regular testing. Renal function tests showed normal sodium, potassium, urea, and creatinine levels. Given the uterine malformation a renal scan of Miss A.'s kidneys was performed at the fetal anatomy scan and no abnormality was detected. Given the normal renal ultrasound result no other renal imaging of the patient's kidneys was performed. The anatomy scan however showed a right multicystic dysplastic kidney in the fetus with no other abnormality (Figure 2). A repeat anatomy scan at 28 weeks showed a shrinking right kidney from 4 cm to 2.5 cm as well as a prominent in size and slightly echogenic left kidney (Figure 3).

At 21 weeks when Miss A. was hospitalized for an unprovoked episode of a minor antepartum hemorrhage, MODY-5 was suspected for the first time given her unexplained early onset diabetes and uterine malformation. Her renal function tests repeated during that hospitalization confirmed the presence of hyperuricemia (0.37 mmol/L) and a hypomagnesaemia of 0.4 mmol/L was discovered. Other biochemical tests were normal.

She was referred to the geneticist for counseling and testing MODY associated gene disorders, which came back positive for a heterozygous deletion of the hepatocyte nuclear factor 1-beta (HNF1B) gene and confirmed the diagnosis. Subsequently pancreas elastase was measured to test exocrine pancreas function which was fortunately normal. The patient's family history is strongly positive for diabetes and renal cysts have been diagnosed in her maternal cousin and her child.

Due to the diabetes diagnosis growth scans of the female fetus were performed every 4 weeks. The fetus remained in breech position during the course of the pregnancy. At 29 + 5 weeks of gestation a polyhydramnios of 31 cm was noted. At 32 weeks the patient presented to our unit with threatened preterm labor and was started on Betamethasone intramuscularly for lung maturation and Nifedipine orally for tocolysis. She was discharged after 48 hours when she had settled. At this point the baby was on the 96th centile of the Australasian Society of Ultrasound in Medicine ASUM charting for growth with an estimated fetal weight of 2.34 kg. The amniotic fluid index had reduced to 21. She represented after 5 days with premature prelabor rupture of membranes and went into spontaneous labor and the baby was delivered vaginally from breech position.

**Figure 4:**
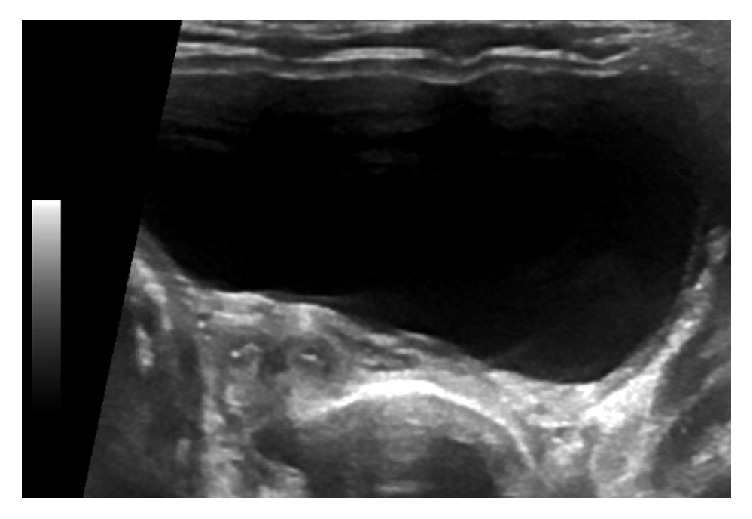
Neonatal bicornuate uterus 1 week postpartum.

The baby girl was born alive, with APGARS 2-3-6, and a birth weight of 2105 g. The physical examination was normal. She was admitted to the neonatal intensive care unit of our hospital where she remained for 5 weeks with an uncomplicated course. However, at one week postpartum a neonatal ultrasound scan confirmed an absent right kidney and the presence of a bicornuate uterus (Figure 4) in the neonate; no other abnormality was seen. The dysplastic right kidney had been completely resorbed. Genetic testing was arranged and showed a heterozygous deletion of the HNF1B gene. An elevated creatinine of 71 umol/L was found in the neonate on laboratory testing. This is attributed to the premature birth as retesting after 6 weeks showed a normal kidney function.

## 3. Discussion

Oram at al. showed that there is a high prevalence of HNF1B mutations in women with both uterine and renal abnormalities. In contrast, no mutations were found in any woman who had an isolated uterine abnormality. They concluded that HNF1B testing should be offered to women who have renal anomalies in addition to uterine anomalies but not routine screening of all women with a uterine abnormality [10]. In our case, mild renal impairment was present through hyperuricemia and hypomagnesaemia even though renal morphologic abnormalities were not detected on ultrasound. We suggest that genetic testing should be offered to young female patients with uterine abnormalities and otherwise unexplained diabetes even in the absence of kidney abnormalities on imaging if kidney impairment is present on laboratory functions.

The diagnosis is important as sometimes monogenic diabetes can be difficult to manage and can lead to microangiopathic complications. Diagnosing the disease in a patient will permit genetic counseling and genetic testing of family members where diagnosis is suspected.

The renal implications of MODY-5 can be variable ranging from very mild impairment with morphologically normal kidneys and renal impairment evident on renal functions tests only [4], like in our patient's case, to end stage renal disease requiring renal replacement therapy.

In our case, serial renal scanning of the fetus showed atrophy of the multidysplastic right kidney which probably implies a progressive loss of function in utero over time compensated by the prominence of the contralateral functioning kidney. Apart from the multicystic dysplastic kidney visualized at the anatomy scan possible implications for the fetus include oligo/anhydramnios in case of kidney function deterioration in utero, IUGR, and low birth weight and neonatal cholestatic jaundice [11]. In view of these potentially severe complications genetic testing in the mother and postnatally in the baby is important to confirm the diagnosis and thus anticipate potential difficulties.

## 4. Conclusion

The optimal care for pregnant patients with MODY-5 due to HNF1B mutations is multidisciplinary and involves obstetricians, endocrinologists, geneticists, nephrologists, and pediatricians. Obstetrician should be aware of this condition due to its possible implications in maternal and fetal morbidity and arrange for genetic testing in patients with early onset diabetes that is not type I diabetes and genitourinary malformations. In our case while the mother's kidneys appear structurally normal on ultrasound an impaired renal function was discovered on further testing. Despite the more common association of MODY-5 with renal malformation we think that genetic testing is also warranted in cases where genital malformations are diagnosed and renal impairment is only present on laboratory testing.
